# Biometric and Structural Ocular Manifestations of Anterior Megalophthalmos

**DOI:** 10.3389/fmed.2022.732452

**Published:** 2022-05-19

**Authors:** Tian-Hui Chen, Ze-Xu Chen, Min Zhang, Jia-Hui Chen, Li-Na Lan, Yongxiang Jiang

**Affiliations:** ^1^Department of Ophthalmology and Vision Science, Eye & ENT Hospital of Fudan University, Shanghai, China; ^2^Key Laboratory of Myopia of State Health Ministry, Shanghai Key Laboratory of Visual Impairment and Restoration, Shanghai, China; ^3^State Key Laboratory of Ophthalmology, Zhongshan Ophthalmic Center, Sun Yat-sen University, Guangzhou, China

**Keywords:** anterior megalophthalmos, ocular manifestations, lens subluxation, cataract, megalocornea

## Abstract

**Objective:**

The aim of this study was to examine the biometric ocular manifestations and structural ocular features of anterior megalophthalmos (AM).

**Methods:**

Fifteen patients with AM (30 eyes) from the Eye & ENT Hospital of Fudan University were included. The age-matched control group consisted of 30 participants (30 eyes) who underwent Pentacam HR and IOLMaster 700 measurements for one normal eye. Data on demographics, biometric manifestations, and genotypes were carefully compared.

**Results:**

A total of 15 patients with AM and 30 control patients were enrolled. There were no differences in age (37.27 ± 19.1 vs. 31.43 ± 19.69 years, *P* = 0.249) between these two groups. AM eyes were characterized by premature cataracts (11/30, 36.67%) and zonular weakness with lens subluxation (22/30, 73.33%) compared with the control group. Notably, 20 of the 30 AM eyes (66.67%) had significant posterior iris bowing, and 16 of the 30 AM eyes (53.33%) showed an enlarged ciliary ring on ultrasound biomicroscopy (UBM). Mean corneal curvature was lower in the AM eyes (42.01 ± 2.06 D vs. 43.14 ± 1.38 D, *P* = 0.023). There was no significant difference in corneal pachymetry and central endothelial cell count between the AM and control groups. Significant differences were found in terms of the anterior chamber and white-to-white (WTW) among the Pentacam HR and IOLMaster 700 in patients with AM (*P* < 0.05). The difference was 0.53 ± 0.48 mm and 0.36 ± 0.14 mm, respectively (*P* < 0.001).

**Conclusion:**

The results of this cohort study conclude the biometric and structural ocular manifestations in Chinese cohorts. Posterior iris bowing (66.67%) and lens subluxation (73.33%) are the most characteristic findings in patients with AM with anatomical abnormalities of megalocornea and a deep anterior chamber, although corneal biometric manifestations of AM included flatter cornea and lower total corneal astigmatism. The knowledge of ocular manifestations of AM is important for diagnosis and preparation for the operation in advance to avoid intraoperative and postoperative complications. Significant differences were found in the anterior chamber and WTW values between the Pentacam HR and IOLMaster 700. Thus, we suggest that various examinations should be carefully considered before determining an AM diagnosis.

## Introduction

Anterior megalophthalmos (AM) is a non-progressive, bilateral congenital enlargement of the anterior segment, first described by Seefeld in 1914 (1). It is characterized by megalocornea (horizontal diameter ≥12.5 mm) with a very deep anterior chamber and enlarged ciliary ring, although corneal histology and thickness remain normal or moderately thin (2). The main causes of decreased vision in patients with AM are premature cataracts at the age of 30–50 years and lens subluxation (3). Posterior segment abnormalities, such as vitreous fibrillar degeneration with liquefaction and retinal detachment, are also found in patients with AM (4). AM is also known as X-linked megalocornea since X-linked recessive inheritance exists in 50% of patients with AM (5). The AM locus maps on the long arm of the X chromosome in the region Xq12-q26 (6). Autosomal transmission is the cause of AM in 40% of the patients, and *de novo* mutations at this locus were found in the remaining 10% of patients (7). *CHRDL1* gene is determined as a virulence gene of AM while Marfan syndrome, Trisomy 21, Apert syndrome, and mucolipidosis type II were found to be associated with AM (8).

Although several challenging cases of cataract surgeries in patients with AM have been reported (3, 9–14), no study has focused on the biometric and structural ocular manifestations of patients with AM with a control group, as AM is a rare and sporadic disease.

To better define the biometric and structural ocular manifestations of patients with AM, we analyzed the ocular findings in 15 patients with AM and compared them to those of the control group.

## Materials and Methods

### Study Population

For a period of 5 years (January 2016 to January 2021), 15 records of patients with AM (30 eyes) and 36 control participants (36 eyes) who presented to the Eye & ENT Hospital of Fudan University were enrolled in this retrospective case-control study. The Institutional Review Board approved this study, with the extension of our randomized controlled trial (ChiCTR2000039132). All participants signed a standard consent form, including consent for data privacy. The medical and family histories of all participants were carefully recorded.

### Patient Selection

Anterior megalophthalmos was diagnosed based on megalocornea (horizontal diameter ≥12.5 mm) with a very deep anterior chamber. Pentacam HR and IOLMaster 700 were used to measure the white-to-white (WTW) distance, and the maximum value was used to diagnose AM. The AM group comprised 15 patients with AM (30 eyes). The control group consisted of 36 participants (36 eyes) who were matched for age and sex to the AM group. Six patients (6 eyes) were excluded because important examination data were missing. None of the participants had a history of ocular trauma or other ocular surgery, uveitis or glaucoma, fundus abnormalities, dry eye disease, or diabetic retinopathy. Patients who wore contact lenses within the previous 2 weeks of examinations were excluded. Patients diagnosed with Marfan syndrome, Trisomy 21, Apert syndrome, and Mucolipidosis type II were excluded from the study.

### Ophthalmological Examination

A slit-lamp microscope examination of the anterior segment was used to identify lens subluxation if the lens edge was clearly visible after pupil dilation. Intraocular pressure (IOP) was measured using non-contact tonometry (NCT; Nidek NT-530, Aichi, Japan). All anterior segment eye photographs were recorded.

### Biometry

Biological characteristics were collected using the Pentacam HR system with a rotating Scheimpflug camera (Pentacam; Oculus, Wetzlar, Germany) and partial coherence interferometry (IOLMaster 500; Carl Zeiss Meditec, Jena, Germany) from January 2016 to March 2018; the IOLMaster 700 (IOLMaster 700; Carl Zeiss Meditec, Jena, Germany) was used from March 2018 to January 2021. All measurements were made at a pupil scan of 6 mm in diameter. We performed ultrasound biomicroscopy (UBM) with a 50 MHz probe (Opko Instrumentation, Miami, FL, United States) and ZS3 Ultrasound System (ZS3 Ultrasound System, Mindray, China) in patients with AM. All ocular findings were carefully recorded, including atrophy of the iris, posterior iris bowing, hypoplasia of the pupil dilator muscle, inadequate pupillary dilatation, early onset cataracts, lens subluxation, enlarged ciliary ring, posterior staphyloma, and retinal breaks.

For each eye, the mean of three repeated measurements was obtained by experienced ophthalmologists.

### Statistical Analyses

All statistical analyses were performed using SPSS 20.0 (IBM Corp., Armonk, NY, United States). Continuous variables are expressed as mean ± SD. The Kolmogorov–Smirnov test was used to confirm the normal distribution of the data. Student’s *t*-test, the Mann–Whitney *U* test, and the chi-square test were used to compare biometric data between the AM and control groups, and the paired *t*-test was used to compare the mean values of the ocular parameters from the two different biometers. To assess the agreement between the two devices, a Bland-Altman analysis was performed by plotting the differences between the measured and average values. The intraclass correlation coefficient (ICC) was used to assess the agreement between individual measurements from two different devices. Statistical significance was set at *P* < 0.05.

## Results

### Study Population

In the study population of 45 adults, 15 [7 (46.67%) women, 8 (53.33%) men] were patients with AM, and 30 [11 (36.67%) women and 19 (63.33%) men] were controls; the mean age was 37.27 ± 19.1 and 31.43 ± 19.69 years, respectively (*P* = 0.249).

### Biometry

The ocular characteristics of the AM and control groups are summarized in Table 1. The IOP in the AM and control eyes was comparable (*P* = 0.512). There was no significant difference in the central endothelial cell count and corneal pachymetry between the AM and control groups. A significant difference in anterior chamber depth (ACD) was observed in the AM group compared with the control group (4.13 ± 1.22 mm vs. 3.17 ± 0.26 mm, *P* < 0.001). The axial length of the AM group was significantly longer than in the controls’ eyes (26.04 ± 3.25 mm vs. 23.33 ± 0.78 mm, *P* < 0.001). The anterior corneal curvature (Km value) and total corneal refractive power were significantly flattered in the AM group than in the control group (*P* = 0.023 and *P* = 0.025, respectively). Further analysis of female and male patients with AM is shown in Table 2. Corneal pachymetry of the female group was thicker than the male group, and flatter cornea was also found in the female group.

**TABLE 1:** Baseline characteristics of the AM and control groups.

	AM group	Control group	*P*-value
Subjects/eyes	15/30	30/30	–
Sex (female: male)	7/8	11/19	0.398
Eyes (right: left)	30 (15/15)	30 (17/13)	0.224
Age (years)	37.27 ± 19.1	31.43 ± 19.69	0.249
WTW (mm)	12.22 ± 0.51	11.8 ± 0.4	0.023*
ACD (mm)	4.13 ± 1.22	3.17 ± 0.26	0.001*
IOP (mmHg)	15.53 ± 3.31	14.94 ± 3.23	0.512
Central ECC (cells/mm^2^)	2795.92 ± 402.77	2866.44 ± 378.18	0.518
Corneal pachymetry (μm)	518.64 ± 47.33	532.36 ± 35.62	0.283
AL (mm)	26.04 ± 3.25	23.33 ± 0.78	<0.001*
KmF (D)	42.01 ± 2.06	43.36 ± 1.18	0.004*
Astig F (D)	1.1 ± 0.59	1.04 ± 0.75	0.77
Km TCRP (D)	42 ± 2.42	43.06 ± 1.26	0.046*
TCA (WFA) (4 mm zone) (D)	−0.79 ± 0.85	−0.87 ± 0.84	<0.001*
WFA Z40 (μm)	0.2 ± 0.11	0.25 ± 0.19	0.243
WFA HO RMS (μm)	0.15 ± 0.06	0.15 ± 0.06	0.883

AM, anterior megalophthalmos; IOP, intraocular pressure; BCVA, best-corrected visual acuity; ECC, endothelial cell count; WTW, white-to-white; LT, lens thickness; AL, axial length; Km, mean keratometry; F, front (anterior corneal surface); Astig, astigmatism; TCRP, total corneal refractive power; ACD, anterior chamber depth; Cornea, corneal diameter (horizontal); TCA (WFA) (4-mm zone), total corneal astigmatism in the 4-mm zone around the corneal apex; WFA Z40, total corneal spherical aberrations (Z4,0) in the 6-mm zone around the corneal apex; WFA HO RMS, root mean square of the total corneal high-order aberrations calculated in the 4-mm zone around the corneal apex. * P < 0.05.

**TABLE 2:** Baseline characteristics of the female and male AM groups.

	Female group	Male group	*P*-value
Subjects/eyes	7/14	8/16	–
Eyes (right: left)	14 (7/7)	16 (8/8)	1
Age (years)	33.81 ± 18.31	41.21 ± 19.88	0.067
WTW (mm)	12.14 ± 0.26	12.29 ± 0.65	0.475
ACD (mm)	4.27 ± 0.62	4.01 ± 1.61	0.573
IOP (mmHg)	15.93 ± 2.9	15.14 ± 3.76	0.386
Central ECC (cells/mm^2^)	2735.08 ± 472.7	2861.83 ± 317.91	0.586
Corneal pachymetry (μm)	540.2 ± 44.61	500.67 ± 43.28	0.029*
AL (mm)	24.73 ± 1.4	27.58 ± 4.13	0.147
Km F (D)	41.06 ± 2.79	43.03 ± 1.45	0.011*
Astig F (D)	1.25 ± 0.92	1.13 ± 0.73	0.682
Km TCRP (D)	40.15 ± 1.76	42.54 ± 1.15	0.001*
TCA (WFA) (4 mm zone) (D)	0.43 ± 0.81	1.15 ± 0.75	0.053
WFA Z40 (μm)	0.18 ± 0.13	0.21 ± 0.1	0.26
WFA HO RMS (μm)	0.14 ± 0.06	0.15 ± 0.06	0.386

AM, anterior megalophthalmos; IOP, Intraocular pressure; BCVA, best-corrected visual acuity; ECC, endothelial cell count; WTW, white-to-white; LT, lens thickness; AL, axial length; Km, mean keratometry; F, front (anterior corneal surface); Astig, astigmatism; TCRP, total corneal refractive power; ACD, anterior chamber depth; Cornea, corneal diameter (horizontal); TCA (WFA) (4-mm zone), total corneal astigmatism in the 4-mm zone around the corneal apex; WFA Z40, total corneal spherical aberrations (Z4,0) in the 6-mm zone around the corneal apex; WFA HO RMS, root mean square of the total corneal high-order aberrations calculated in the 4-mm zone around the corneal apex. * P < 0.05.

### Structural Findings

**FIGURE 1:**
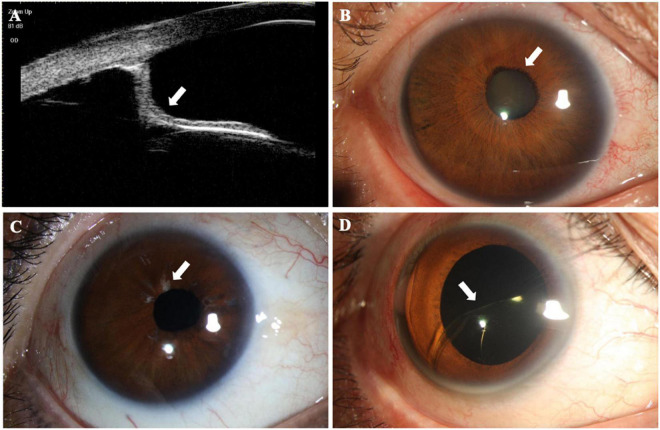
Structural findings in patients with anterior megalophthalmos (AM). **(A)** Ultrasound biomicroscopy image performed through the right eye of an AM patient showing posterior iris bowing (white arrowheads) and a greatly enlarged anterior segment. **(B)** Early onset mature cataract (white arrowheads) was shown in a 29-year-old AM patient with a greater corneal diameter. **(C)** The right eye of a patient with AM showed obvious atrophy of iris (white arrowheads). **(D)** Intraocular lens (IOL) dislocation (white arrowheads) was present in a patient with AM after normal cataract surgery.

The structural findings are summarized in Table 3 and are presented in Figure 1. Since a person may have multiple symptoms, a patient may be counted multiple times when calculating the number of people with symptoms. Atrophy of the iris was documented in 11 of the 30 (36.67%) AM eyes. Posterior iris bowing also had a high frequency (66.67%) in AM eyes indicated from UBM examination. A total of 6.67% of the AM eyes showed hypoplasia of the pupil dilator muscle, and inadequate pupillary dilatation was apparent in 13.33% patients with AM with 0.1 ml 0.5% tropicamide drops 30 min before the cataract surgery. The patients with AM had significant nuclear cataracts with the mean age of 46.91 years in 36.67%, and lens subluxation appeared in 73.33%. Retinal breaks were found only in 3.33% of patients with AM eyes.

**TABLE 3:** Structural ocular findings in patients with AM.

Ocular structure	Pathology	AM eyes
Anterior chamber	Deep anterior chamber	30/30 (100%)
Iris	Atrophy of the iris	11/30 (36.67%)
	Posterior iris bowing	20/30 (66.67%)
Pupil	Hypoplasia of the pupil dilator muscle	2/30 (6.67%)
	Inadequate pupillary dilatation	4/30 (13.33%)
Lens	Early onset cataracts	11/30 (36.67%)
	Lens subluxation	22/30 (73.33%)
Ciliary ring	Enlarged ciliary ring	16/30 (53.33%)
Posterior segment	Posterior staphyloma	9/30 (30%)
	Retinal breaks	1/30 (3.33%)

*AM, anterior megalophthalmos.*

It should be noted that there was 1 patient who complained of intraocular lens (IOL) dislocation at the initial visits was excluded from the AM group due to his previous ocular surgery; however, he was eventually diagnosed with AM after a series of ocular examinations.

### Assessment Agreement for Patients With Anterior Megalophthalmos

**FIGURE 2:**
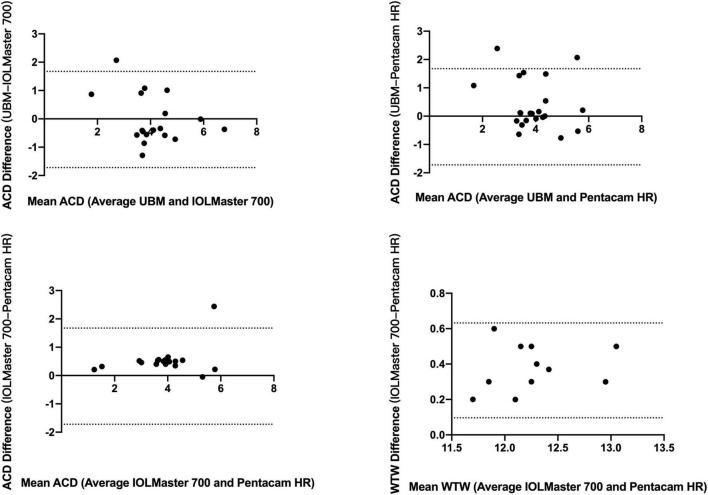
Bland-Altman plots of comparison between ACD and WTW by UBM, IOLMaster 700, and Pentacam HR. ACD, anterior chamber depth; WTW, white-to-white.

Table 4 shows the mean values of ACD and WTW obtained using different devices. The Friedman test revealed a statistically significant difference when ACD measurements were compared between the UBM, IOLMaster 700, and Pentacam HR (*P* = 0.001). Specifically, after using the Quade *post hoc* test, significant differences were found between UBM vs. IOLMaster 700, UBM vs. Pentacam HR, and IOLMaster vs. Pentacam HR (*P* < 0.001, all). The different Bland-Altman plots are provided in Figure 2 for the 3 possible comparisons between 2 devices, namely, UBM vs. IOLMaster 700, UBM vs. Pentacam HR, and IOLMaster vs. Pentacam HR.

**TABLE 4:** WTW and ACD measurements for different devices.

	IOLMaster 700	Pentacam HR	UBM	*P*-value
ACD (mm)	4.15 ± 1.22	3.74 ± 1.09	4.18 ± 0.96	0.009
WTW (mm)	12.3 ± 0.5	12.24 ± 0.47	NA	<0.001*

	**UBM vs. IOLMaster ICC (95% CI)**	**UBM vs. Pentacam ICC (95% CI)**	**IOLMaster vs. Pentacam ICC (95% CI)**	

ACD (mm)	0.74 (0.43, 0.9)	0.624 (0.28, 0.82)	0.832 (0.17, 0.95)	
WTW	NA	NA	0.680 (−0.53, 0.96)	

ACD, anterior chamber depth; WTW, white-to-white; ICC, intraclass correlation coefficient. *P < 0.05.

Agreement among devices, assessed by the ICC, is shown in Table 4. Only the ACD reached a high ICC (>0.75) between the IOLMaster 700 and Pentacam HR.

## Discussion

Anterior megalophthalmos is a hereditary disease that affects the anterior segment of the eye. The enlarged cornea of AM may be mistaken for congenital glaucoma; however, IOP and corneal clarity are normal in patients with AM (15). However, a review showed that only 3 out of 57 patients (5.26%) presented with glaucoma (2). AM can also be easily confused with megalocornea that has no other ocular abnormalities, excluding corneal enlargement. There is a need to distinguish AM from other developmental abnormalities characterized by corneal enlargement by biometric and structural ocular manifestations.

In simple megalocornea, there are no other ocular abnormalities, excluding corneal enlargement. In patients with AM, the cornea is enlarged, typically with increased ACD. Meire and Delleman (5) reported that axial length was increased in patients with AM, which was also found in our study with a median axial length of 26.04 ± 3.25 mm. Corneal endothelial cells have a normal morphology and cell density, which suggests that corneal endothelial cell hyperplasia occurs concurrently with excessive corneal growth.

Some special pathological changes in patients with AM have been reported in this study, including iris atrophy (36.67%), posterior iris bowing (66.67%), and enlarged ciliary ring (53.33%). AM is a non-progressive condition as lens dislocation (73.33%) and premature development of lens opacities (36.67%) were the most intriguing features of AM observed in this study. Further eye growth with age is not progressively abnormal. With the development of early cataract and lens subluxation, this could be considered a progression of the constellation of features, but AL remains stable after entering adulthood. Additionally, there are several challenges in cataract surgery for patients with AM caused by deep anterior chambers, zonular anomalies, and large capsular bags (7). Many intraoperative and postoperative complications have been reported, such as vitreous loss, lens subluxation/luxation, retinal detachment, and IOL dislocation (16).

Intraocular lens instability related to an oversized capsular bag and zonular anomalies is the highest concern for ophthalmologists. Implantation of IOLs in lens capsule bags or in the ciliary sulcus is only appropriate in cases without significant zonular weakness. In reviewing the literature on AM, different approaches have been applied to avoid IOL decentration (9). Sharan and Billson (11) prescribed aphakic and aphakic contact lenses to patients for visual rehabilitation. Implantation of large custom-made IOLs to ensure endocapsular fixation in problematic capsular bags was reported by Vaz and Osher (17). However, due to high expenses, custom-made IOLs are difficult to obtain. Custom-made IOLs handle the problem of oversized capsular bags while still having a high risk of being dislocated due to 360° zonular weakness caused by an enlarged ciliary ring (13, 16).

To manage insufficient capsular support, iris-claw IOLs have started to be noticed in patients with AM. Retro-pupillary iris-claw IOLs have been proposed as excellent alternatives for patients with AM (18). Compared with sutured anterior chamber IOLs, retropupillary iris-claw IOLs enclavated to the iris are supposed to reduce the potential for complications associated with anterior chamber IOLs, such as corneal endothelial damage, anterior synechia, and glaucoma (10). In recent research, anterior chamber and PC iris-claw IOL fixation were equally effective and safe in eyes with inadequate capsular support (11). Retropupillary iris-claw IOLs also have some disadvantages. The enlarged incision may result in unpredictable astigmatism after surgery. Meanwhile, iridoleptynsis is a common symptom observed in patients with AM, although the additional development of iris lesions may occur after suturing the iris claw (11).

In this study, we tested the agreement of optical biometers in measuring the ACD and WTW in the eyes of patients with AM, as we found a large gap among the different biometers. After comparing the ACD measured by three optical biometers in patients with AM, the results showed that ACD measured by Pentacam HR was significantly lower than that measured by IOLMaster 700 and UBM. A significant difference was also found between the Pentacam HR and IOL Master 700 in WTW. WTW measured by the IOL Master 700 was obviously higher than that measured by the Pentacam HR, indicating that Pentacam HR and IOL Master 700 could not be used interchangeably for measuring WTW. Both devices used anterior segment photography to obtain WTW measurements. The accuracy of WTW measurement depends on algorithms for limbus edge detection and on the light source of the device, as well as the quality of the image captured by optical biometers. It is noted that the WTW value is required in the diagnosis of AM and is also required for choices of IOL implantation methods, including IOL implantation in the ciliary sulcus, in capsular bags, and in *trans*-sclera suture fixation. Therefore, to ensure safety, the maximum value of WTW was used to diagnose AM. Since the calculated power can vary depending on WTW and ACD measurements, it should be carefully considered before cataract surgery for patients with AM.

X-linked recessive inheritance is the most common form of AM, which was found in 50% of AM cases, while the autosomal transmission was observed in 40% of patients with AM, and sporadic transmission was observed in the remaining 10% (19). Gene linkage studies have suggested that the AM locus maps in the region Xq12-q26.9, and men account for 90% of AM cases (8). However, in our series of 15 patients with AM, there were 7 women and 8 men. Homozygous mutations in CPAMD8 in 3 of 5 AM unrelated patients were identified by whole-exome sequencing (Table 5). As a candidate gene for cataract development, CPAMD8 was mapped to chromosome 19p13.2–p13.3, spanning approximately 130 kb. Wiggs (20) identified mutations in CPAMD8 associated with anterior segment dysgenesis. Affected patients showed similar features, including a bilateral enlarged corneal diameter, ectopia lentis, and premature cataract, which matched the patients with AM (21).

**TABLE 5:** The overall spectrum of *CPAMD8* variants found in this study.

Sample	Gene	Variants	Feature	Effect	MutationTaster	ACMG	Heredity
EENT01	CPAMD8	c.4825C > T	New	Missense	Disease causing	Likely pathogenic	AR
	CPAMD8	c.3226G > A	New	Missense	Disease causing	VUS	AR
EENT02	CPAMD8	c.4825C > T	New	Missense	Disease causing	VUS	AR
	CPAMD8	c.3955G > C	New	Missense	Disease causing	VUS	AR
EENT03	CPAMD8	c.3349C > T	New	Missense	Polymorphism	VUS	AR
	CPAMD8	c.949_950delAC	New	Frameshift-deletion	Disease causing	VUS	AR

This study has several limitations. First, as AM is a rare disease, only 15 patients with AM (30 eyes) were collected for a period of 5 years (January 2016 to January 2021) with 14,000 cataract surgeries per year in the study hospital. Only three unrelated female patients underwent whole-exome sequencing. Larger studies of patients with AM may be necessary in the future. Second, because the IOLMaster 700 was used from March 2018 to January 2021, some ocular characteristics measured by IOLMaster 700 were not documented before March 2018; therefore, we excluded patients with missing data when assessing agreement of the IOLMaster 700 and Pentacam HR in patients with AM. Third, a longitudinal study is needed to record postoperative complications and visual acuity in patients with AM.

## Conclusion

Corneal biometric manifestations of patients with AM included flatter cornea, lower total corneal astigmatism, and longer axial length, although corneal morphology and thickness remained normal or moderately thin. Posterior iris bowing, lens subluxation, and very deep anterior chambers are the most characteristic findings in AM. The ACD and WTW values measured by the Pentacam HR were significantly lower than those measured by the IOLMaster 700. Thus, we suggest that various examinations should be carefully considered before determining an AM diagnosis.

## Data Availability Statement

The raw data supporting the conclusions of this article will be made available by the authors, without undue reservation.

## Ethics Statement

The Institutional Review Board approved the study with the extension of our randomized controlled trial (ChiCTR2000039132). All the patients were signed the standard consent form including consent for data privacy.

## Author Contributions

YJ and T-HC were responsible for the research design of the article. MZ and J-HC helped to collect the data of followed MFS patients. T-HC and Z-XC was responsible for statistical analyses. All authors reviewed and revised the final manuscript.

## Conflict of Interest

The authors declare that the research was conducted in the absence of any commercial or financial relationships that could be construed as a potential conflict of interest. The reviewer, JH declared a shared affiliation with the authors to the handling editor at the time of the review.

## Publisher’s Note

All claims expressed in this article are solely those of the authors and do not necessarily represent those of their affiliated organizations, or those of the publisher, the editors and the reviewers. Any product that may be evaluated in this article, or claim that may be made by its manufacturer, is not guaranteed or endorsed by the publisher.
